# PROMISING POLICY AND PRACTICE SUPPORTING FAMILY CAREGIVERS AND RECOMMENDATIONS FOR THE FUTURE

**DOI:** 10.1093/geroni/igad104.0795

**Published:** 2023-12-21

**Authors:** Susan Reinhard, Selena Caldera, Ari Houser, Rita Choula

**Affiliations:** AARP, Washington, District of Columbia, United States; AARP, Washington, District of Columbia, United States; AARP Public Policy Institute, Washington, District of Columbia, United States; AARP, Washington, District of Columbia, United States

## Abstract

There have been significant federal and state policy developments since the 2019 update of Valuing the Invaluable. Many inroads have been made in recognizing and supporting family caregivers, but additional steps can be taken at the federal and state levels, and even in the private sector. The National Strategy to Support Family Caregivers, released in September 2022, lays out a unified approach in five organizing goals to achieving this improvement in support for family caregivers. The policy and practice recommendations highlighted in the Valuing the Invaluable: 2023 Update reflect the National Strategy goals. In this presentation, recent policy changes will be summarized that support caregivers and their care recipients in the health care and LTSS systems, which provide financial relief for caregiving, and help working caregivers stay in the workforce. Then the increasing visibility of the family caregiving experience in media and the arts will be explored. This visibility is essential for bringing broader attention to all aspects of caregiving, normalizing the care experience, and eliminating stigma around disability and dementia. Lastly, policy recommendations for building on the National Strategy and bolstering support for family caregivers and care recipients will be detailed for discussion.

